# Expression, purification and crystallization of CTB-MPR, a candidate mucosal vaccine component against HIV-1

**DOI:** 10.1107/S2052252514014900

**Published:** 2014-08-20

**Authors:** Ho-Hsien Lee, Irene Cherni, HongQi Yu, Raimund Fromme, Jeffrey D. Doran, Ingo Grotjohann, Michele Mittman, Shibom Basu, Arpan Deb, Katerina Dörner, Andrew Aquila, Anton Barty, Sébastien Boutet, Henry N. Chapman, R. Bruce Doak, Mark S. Hunter, Daniel James, Richard A. Kirian, Christopher Kupitz, Robert M. Lawrence, Haiguang Liu, Karol Nass, Ilme Schlichting, Kevin E. Schmidt, M. Marvin Seibert, Robert L. Shoeman, John C. H. Spence, Francesco Stellato, Uwe Weierstall, Garth J. Williams, Chunhong Yoon, Dingjie Wang, Nadia A. Zatsepin, Brenda G. Hogue, Nobuyuki Matoba, Petra Fromme, Tsafrir S. Mor

**Affiliations:** a Department of Chemistry and Biochemistry, Arizona State University, PO Box 871604, Tempe, AZ 85287-1604, USA; b School of Life Sciences, Arizona State University, PO Box 874501, Tempe, AZ 85287-4501, USA; c Center for Infectious Diseases and Vaccinology, Biodesign Institute, Arizona State University, PO Box 874501, Tempe, AZ 85287-5401, USA; d Center for Free-Electron Laser Science, DESY, Notkestrasse 85, 22607 Hamburg, Germany; e Linac Coherent Light Source, SLAC National Accelerator Laboratory, 2575 Sand Hill Road, Menlo Park, CA 94025, USA; f University of Hamburg, Luruper Chaussee 149, 22761 Hamburg, Germany; g Department of Physics, Arizona State University, PO Box 871504, Tempe, AZ 85287-1504, USA; h Max-Planck-Institut für medizinische Forschung, Jahnstrasse 29, 69120 Heidelberg, Germany; i European XFEL GmbH, Albert-Einstein-Ring 19, 22761 Hamburg, Germany

**Keywords:** X-ray crystallography, femtosecond nanocrystallography, HIV-1, gp41, membrane-proximal region, cholera toxin B subunit, crystallization, free-electron lasers

## Abstract

CTB-MPR is a fusion protein between the B subunit of cholera toxin (CTB) and the membrane-proximal region of gp41 (MPR), the transmembrane envelope protein of *Human immunodeficiency virus 1* (HIV-1), and has previously been shown to induce the production of anti-HIV-1 antibodies with antiviral functions. To further improve the design of this candidate vaccine, X-ray crystallography experiments were performed to obtain structural information about this fusion protein. Several variants of CTB-MPR were designed, constructed and recombinantly expressed in *Escherichia coli*. The first variant contained a flexible GPGP linker between CTB and MPR, and yielded crystals that diffracted to a resolution of 2.3 Å, but only the CTB region was detected in the electron-density map. A second variant, in which the CTB was directly attached to MPR, was shown to destabilize pentamer formation. A third construct containing a polyalanine linker between CTB and MPR proved to stabilize the pentameric form of the protein during purification. The purification procedure was shown to produce a homogeneously pure and monodisperse sample for crystallization. Initial crystallization experiments led to pseudo-crystals which were ordered in only two dimensions and were disordered in the third dimension. Nanocrystals obtained using the same precipitant showed promising X-ray diffraction to 5 Å resolution in femtosecond nanocrystallography experiments at the Linac Coherent Light Source at the SLAC National Accelerator Laboratory. The results demonstrate the utility of femtosecond X-ray crystallography to enable structural analysis based on nano/microcrystals of a protein for which no macroscopic crystals ordered in three dimensions have been observed before.

## Abstract 2

Femtosecond X-ray crystallography allows structural analysis of a difficult-to-crystallize fusion protein that is a potential component of a candidate HIV-1 subunit vaccine.

## 1. Introduction   

**Figure 1:**
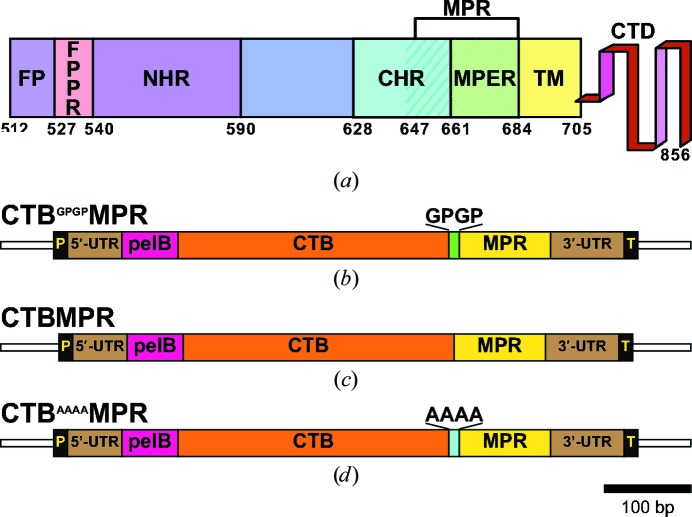
(*a*) The architecture of gp41. FP (residues 512–527), fusion peptide; FPPR (residues 528–539), fusion peptide proximal region; NHR (residues 540–­590), N-terminal heptad-repeat region; CHR (residues 628–661), C-­terminal heptad-repeat region; MPER (residues 662–684), membrane-proximal external region; MPR (residues 647–684, hatched), membrane-proximal region; TM (residues 685–705), transmembrane domain; CTD (residues 706–856), cytoplasmic C-terminal domain. (*b*, *c*, *d*) DNA constructs for the expression in *E. coli* of the indicated CTB-MPR fusion proteins are based on elements of the pET-22b expression vector. P, T7 bacteriophage promoter; 5′-UTR, upstream untranslated region; pelB, the periplasmic targeting sequence of pectate lyase B of *Erwinia carotovora*; CTB, cholera toxin B subunit; MPR, the membrane-proximal region of the gp41 protein of HIV-1; 3′-UTR, downstream untranslated region; T, T7 terminator. The GPGP and AAAA linkers are indicated above their respective constructs. The three constructs encode the fusion proteins CTB^GPGP^MPR (*b*), CTBMPR (*c*) and CTB^AAAA^MPR (*d*) with expected molecular masses (after the processing of the pelB leader sequence) of 16.7, 16.4 and 16.7 kDa, respectively.

The envelope glycoprotein of HIV-1 is a complex composed of three copies of a heterodimer consisting of gp120 and gp41. The latter (Fig. 1 ▶ *a*) is embedded in the viral membrane, mediates the fusion between viral and cellular membranes (Teixeira *et al.*, 2011 ▶) and plays a major role in viral transmission across the epithelial barrier (Shen *et al.*, 2010 ▶; Bomsel *et al.*, 2011 ▶; Hessell *et al.*, 2010 ▶; Tudor *et al.*, 2009 ▶). Mucosal transmission of HIV-1 through monostratified epithelia depends on interactions between the viral envelope membrane protein gp41 and the glycolipid galactosyl ceramide (GalCer) on epithelial cells (Alfsen *et al.*, 2001 ▶; Alfsen & Bomsel, 2002 ▶; Meng *et al.*, 2002 ▶), and also on dendritic cells, the most important class of antigen-presenting cells (Bomsel & Magérus-Chatinet, 2004 ▶; Magérus-Chatinet *et al.*, 2007 ▶). The GalCer binding domain of gp41 is mediated by a highly conserved membrane-proximal region (MPR) of gp41 consisting of residues 649–684. This region of the protein spans the membrane-proximal external region (MPER; residues 660–683; reviewed by Zwick, 2005 ▶), which includes the epitopes for the broadly neutralizing and transcytosis-blocking monoclonal human antibodies 2F5, 4E10 and 10E8 (Zwick *et al.*, 2001 ▶; Huang *et al.*, 2012 ▶) and, unlike the MPER itself (residues 650–683), maintains important structural and functional attributes of the native protein, including oligomerization and GalCer binding (Alfsen & Bomsel, 2002 ▶).

An effective vaccine against HIV-1 should ideally consist of components that target multiple steps of the viral trans­mission/infection process. Most importantly, it should engage the virus early in the cycle to minimize the chance of establishing viral reservoirs and subsequent re-dissemination (Valdiserri *et al.*, 2003 ▶). From a worldwide perspective, HIV-1 transmission most commonly occurs through the exposure of mucosal surfaces to HIV-positive secretions (Pope & Haase, 2003 ▶; Hladik & McElrath, 2008 ▶; Haase, 2011 ▶). Therefore, the crucial involvement of the MPR in mucosal transmission of HIV and the well characterized, albeit rare, antiviral immune responses directed against this domain make it a prime candidate for an active vaccine.

However, by itself, the MPR was shown to act as a rather poor immunogen and was sensitive to its structural context (Denner, 2011 ▶). To overcome these limitations and in particular to boost immunogenicity at the mucosal surface, we have been exploring the MPR through its fusion to the mucosa-targeting cholera toxin B subunit (CTB; Matoba *et al.*, 2004 ▶, 2006 ▶, 2008 ▶, 2009 ▶). The CTB pentamer is taken up by mucosal immune cells through endocytosis mediated by binding to G_M1_ gangliosides (Merritt *et al.*, 1994 ▶). Thus, a fusion protein comprised of CTB and MPR provides the target epitopes needed to elicit anti-HIV-1 antibodies directed at the MPR and combines the mucosal targeting of CTB and its immunogenicity. However, anti-MPR responses elicited by CTB-MPR were not optimal and indicated a need for an improved MPR-based immunogen (Matoba *et al.*, 2004 ▶, 2006 ▶, 2008 ▶, 2009 ▶, 2011 ▶).

Understanding the function of MPR and the membrane-associated processes it takes part in, such as transcytosis and membrane fusion, as well as its interactions with the immune system, requires knowledge of its structure. To better understand the immunogenicity of the fusion protein and to enable us to design even more immunogenic MPR fusion proteins, we turned to structural investigation. Here, we report on the expression of several novel variants of CTB-MPR with different linkers between the two fusion partners. We further report the purification of these proteins and their biochemical characterization, as well as initial crystallization experiments and X-ray crystallographic analysis.

## 2. Materials and methods   

### 2.1. Vectors for bacterial expression of CTB-MPR fusion protein variants   

The expression vectors used in this study were all based on the *Escherichia coli* periplasmic targeting vector pET-22b(−) (Novagen; Figs. 1 ▶ *b*, 1 ▶ *c* and 1 ▶ *d*). The cloning of a synthetic gene encoding a fusion protein comprising CTB and the MPR with a flexible GPGP linker between them to obtain the plasmid pTM101 has been described previously (Matoba *et al.*, 2004 ▶). To obtain a fusion protein without the C-terminal His tag engineered on the protein product of pTM101, we PCR-amplified the coding sequence with primers oTM066 and oTM123 (see Table 1 ▶ for a complete list of the oligonucleotides used in this work), and following digestion with *Nco*I and *Blp*I cloned them into the respective sites in the pET-22b(−) vector to obtain pTM199. In this work, the fusion-protein product of this vector is called CTB^GPGP^MPR.

The plasmid pTM199 served as the template to construct two additional variants of the fusion protein by overlap PCR (Aiyar *et al.*, 1996 ▶). Briefly, in two separate PCR reactions, the two ‘end’ primers oTM066 and oTM123 were used, respectively, with two ‘mutagenizing’ primers oTM469 and oTM468 to amplify two partially overlapping fragments of the coding region of the fusion gene. The two fragments, now containing the deleted linker region, were gel-purified and used together as templates with the ‘end’ primers to PCR-amplify the complete fusion gene. The fragment was cloned into a pTOPO-TA vector (Invitrogen) to yield pTM545, and the correct sequence was verified. An *Nco*I–*Blp*I fragment from pTM545 was cloned into the corresponding sites of a pET-26b(+) vector to yield pTM556. The periplasmic-directed, linker-less version of the fusion protein encoded by this vector is referred to here as CTBMPR. A similar strategy (employing the ‘end’ primers oTM066 and oTM123 together with the ‘mutagenizing’ primers oTM522 and oTM521) was used to create a vector, pTM646, encoding a variant fusion protein with a tetra-alanine linker dubbed CTB^AAAA^MPR.

### 2.2. Expression and purification of fusion-protein variants   

Bacterial expression of CTB-MPR fusion-protein variants followed our previously published protocol for the CTB^GPGP^MPR variant (Matoba *et al.*, 2008 ▶). Similarly, we have modified the previously published purification protocol (Matoba *et al.*, 2008 ▶) to avoid precipitation of the protein at high pH and to replace the previously used detergents with detergents that would be compatible with crystallization. Briefly, cell pellets from 2 l culture (approximately 5 g) were resuspended in 20 ml ice-cold phosphate-buffered saline (PBS; 137 m*M* NaCl, 2.7 m*M* KCl, 10 m*M* Na_2_HPO_4_, 1.8 m*M* KH_2_PO_4_) containing 1 m*M* phenylmethanesulfonyl fluoride (PMSF), a serine protease inhibitor, to prevent protein degradation. The cells were lysed by passing them twice through a microfluidizer (Microfluidics Microfluidizer) with PMSF added again after the first pass. The lysate was collected in a 40 ml Oak Ridge tube and was centrifuged at 36 000*g* for 20 min. The insoluble fraction was washed once by repeated resuspension (in 30 ml ice-cold PBS) and centrifugation. If not immediately used, the pellet was frozen at −80°C.

The pellet, containing the membrane fraction, was resuspended in 30 ml buffer (20 m*M* bicine pH 8.0, 500 m*M* NaCl). To fully homogenize the solution, the sample was sonicated at 20% amplitude in 30 s runs (Model 300V/T Ultrasonic Homogenizer, Biologics Inc.) until a homogenous turbid suspension was obtained. The detergent *n*-dodecyl-β-d-maltoside (βDDM) was used for solubilization. A stock solution of 10%(*w*/*v*) was added to a final concentration of 1%(*w*/*v*). The protein was solubilized at 4°C overnight with agitation.

The protein solution was centrifuged at 36 000*g* for 20 min and the pellet was discarded. A gravity-driven column (Bio-Rad Econo-Column) containing cobalt affinity resin (40 ml bed volume; Talon, Clontech) was equilibrated with binding buffer (resuspension buffer supplemented with 0.05% βDDM). The sample was then loaded onto the column and washed with six bed volumes of binding buffer and ten bed volumes of wash buffer (20 m*M* bicine pH 8.0, 50 m*M* NaCl, 5 m*M* imidazole, 0.05% βDDM) to remove weakly bound proteins. Tightly bound proteins were eluted by the application of three bed volumes of elution buffer (20 m*M* bicine pH 8.0, 50 m*M* NaCl, 150 m*M* imidazole, 0.05% βDDM).

The eluted fractions were pooled and then concentrated to approximately 2 mg ml^−1^ using 50 kDa molecular-weight cutoff (MWCO) concentrators (Vivaspin 20 VS2031, Sartorius Stedim Biotech). Concentrated samples were further purified by size-exclusion chromatography (SEC; Superdex 200, GE Healthcare; column volume 24 ml, fluid phase 8 ml) using a high-pressure liquid-chromatography instrument (HPLC; ÄKTAexplorer, Pharmacia). The running buffer consisted of 20 m*M* HEPES pH 7.5, 10 m*M* CaCl_2_, 0.02% βDDM. For analytical separations, a sample (200 µl) of concentrated CTB-MPR variant was loaded onto the SEC column and chromatography was performed at a flow rate of 0.5 ml min^−1^. The column was loaded with a maximum of 1 ml sample for preparative separation runs, with only slight broadening of the peaks being observed. The protein elution was detected by absorption at 280 nm. Fractions corresponding to individual peaks were collected and pooled.

The concentrations of CTB-MPR variant preparations were determined spectrophotometrically (*A*_280_) using ∊_280_ = 39 380 *M* ^−1^ cm^−1^ (∊_280_ was calculated with the *ProtParam* web application; http://web.expasy.org/protparam/). Assembly of pentamers of the CTB-MPR variants was monitored by ELISA using G_M1_ gangliosides for capture and the MPR-specific human monoclonal antibody 2F5 as described previously (Matoba *et al.*, 2008 ▶) and by nondenaturing SDS–PAGE (see below).

### 2.3. SDS–PAGE and immunoblotting   

SDS–PAGE using tricine-based buffers in a Bio-Rad Mini-PROTEAN Tetra Cell was performed as previously described by Lawrence *et al.* (2011 ▶) based on the method of Schägger (2006 ▶). Following electrophoresis, the gels were stained with Coomassie Brilliant Blue, subjected to silver staining (Lawrence *et al.*, 2011 ▶) or processed for immunoblotting.

For immunoblotting, the acrylamide gel was rinsed with water and equilibrated in anode buffer consisting of 60 m*M* Tris, 40 m*M* *N*-cyclohexyl-3-aminopropanesulfonic acid (CAPS), 15% methanol. The PVDF membrane was prepared by soaking in 100% methanol and then equilibrated in cathode buffer consisting of 60 m*M* Tris, 40 m*M* CAPS, 0.1% SDS. The gel and the membrane were sandwiched between extra-thick blot filter papers (Bio-Rad) soaked in the appropriate electrode buffer and proteins were electroblotted for 30 min at 120 mA (Bio-Rad Transfer-blot SD Semi-dry Transfer Cell). Following blocking for 1 h in PBSTM (PBS, 0.05% Tween 20, 5% dry milk), the PVDF membrane was further incubated in the presence of the 2F5 monoclonal antibody (kindly provided by the NIH’s AIDS Reagent Program; 1:10 000 dilution; Purtscher *et al.*, 1996 ▶). The membrane was then washed for 3 × 30 min in PBST (PBS, 0.05% Tween 20) prior to incubation (1 h) with rabbit anti-human IgG conjugated to horseradish peroxidase (1:20 000 dilution in PBSTM; Santa Cruz Biotechnology sc-2923). Following three additional 30 min washes, the PVDF membrane was then soaked with Bio-Rad Clarity Western ECL substrate solution and imaged with a UVP BioSpectrum 500C Imaging System.

CTB forms a very stable pentamer that resists dissociation by SDS in a monomer concentration-dependent manner. Nonetheless, CTB pentamers can be denatured by heat and by reduction of the intermolecular disulfide bridges that stabilize the oligomers (Zrimi *et al.*, 2010 ▶; Yasuda *et al.*, 1998 ▶). Nondenaturing SDS–PAGE was conducted as described above except that DTT was omitted from the loading buffer and the samples were not boiled prior to loading them onto gels (Matoba *et al.*, 2008 ▶).

### 2.4. Dynamic light scattering   

Dynamic light-scattering (DLS) measurements were performed using a NaBiTec GmbH setup comprising a SpectroSize 302 (Molecular Dimensions) in combination with an S6D microscope (Leica). The purified protein sample (concentrated to 8 mg ml^−1^ as described above) was illuminated in a 3 µl hanging drop using a 24-well crystallization plate (VDX Greased Plate, Hampton Research) covered with siliconized-glass circular cover slides (22 mm; Hampton Research). The well itself was filled with 600 µl SEC running buffer. Prior to the measurement, the protein solution was centrifuged (1000*g*, 30 min, 4°C) to remove possible dust particles. During the measurement, the temperature was set to 20°C. Ten consecutive measurements, each with an integration time of 20 s, were averaged. An estimate of the hydrodynamic size was obtained with the instrument software using the following parameters: refractive index 1.33, viscosity 1.006, shape factor 1.0, hydrated shell 0.2 nm.

### 2.5. Crystallization experiments   

For crystallization experiments, the fusion-protein preparations were concentrated to a final concentration of 10 mg ml^−1^ using 100 kDa MWCO concentrators (Amicon Centricon YM-100). Initial broad screening for crystallization conditions used NeXtal crystallization kits (The PEGs Suite, The MBClass Suite and The MBClass II Suite) with the vapor-diffusion technique. Screening was performed using 96-well plates (Qiagen CrystalEX 96-well Conical Flat Plate) with the sitting-drop method, where each reservoir well contained 100 µl precipitant solution. The purified protein solution was then mixed in a 1:1 ratio (1 µl:1 µl) with the reservoir solution in the sitting-drop well.

Conditions that produced crystals served to guide us in fine screening by the hanging-drop method using 24-well plates (Hampton Research VDX Greased Plates), with each reservoir well containing 900 µl precipitant solution. The purified protein solution was then mixed with the reservoir solution (3 µl each) on a siliconized glass circle cover slide (22 mm; Hampton Research) and the slide was used to seal the well.

As the broad screening produced crystals in the presence of polyethylene glycol (PEG), our fine screens centered on the addition of PEGs of various defined chain lengths (molecular weights ranging from 300 to 4000) under pH, salt and ionic strength conditions that produced crystals that were hexagonal from one viewing plane and completely round as viewed perpendicularly. Specifically, combinatorial screens involved testing various buffers (50 m*M* of either sodium acetate pH 4.6, MES pH 6.5 or HEPES pH 7.5) and salts (100 m*M* of either NH_4_Cl, NaCl, CaCl_2_ or MgCl_2_).

Fine screens for optimal crystallization conditions of CTB^GPGP^MPR were conducted with 0.1 *M* HEPES pH 7.5 and varying concentrations of PEG 400. The best crystals appeared using a reservoir solution consisting of 34% PEG 400, 0.2 *M* BaCl_2_, 20% ethylene glycol. The hanging drop contained 1.5 µl reservoir solution, 0.5 µl 2 *M* ammonium acetate, 2 µl protein sample and 0.41 µl 10% CYMAL-4 (yielding a final concentration of 0.74% or 2× the critical micelle concentration).

Fine screens for optimal crystallization conditions of CTBMPR were conducted with the choice buffer (50 m*M* HEPES pH 7.5) and focused on varying concentrations of choice PEGs (20–40% PEG 300, 5–20% PEG 3000 or 5–20% PEG 4000) in the presence of 100 m*M* NH_4_Cl, NaCl or CaCl_2_. In parallel, we conducted salt-concentration screens (50–200 m*M*) for NH_4_Cl, NaCl and CaCl_2_ in solutions that contained either 25% PEG 300, 10% PEG 3000 or 10% PEG 4000. Finally, under the choice conditions of buffer, PEG and salt (50 m*M* HEPES pH 7.5, 25% PEG 300, 200 m*M* NH_4_Cl) we conducted an additive screen (Hampton Research Additive Screen), in which 96 different additives were added (1 µl) to the individual drop well in a Qiagen CrystalEX 96-well Conical Flat Plate along with the protein and reservoir drop mixture, which consisted of 50 m*M* HEPES pH 7.5, 20% PEG 300, 10%(*w*/*v*) either glycerol, 2-propanol or CYMAL-4 and 200 m*M* salt (either NH_4_Cl, NaCl or CaCl_2_).

Fine screens for optimal CTB^AAAA^MPR crystallization conditions were performed with 100 m*M* Tris pH 8.5 or 50 m*M* HEPES pH 7.5 while varying the concentrations of either PEG 1000 (10–30%) or PEG 3350 (5–20%) in the presence of 200 m*M* of either NH_4_Cl, NaCl, CaCl_2_ or NH_4_HCO_2_. In parallel, salt-concentration screens of NH_4_Cl, NaCl, CaCl_2_ and NH_4_HCO_2_ from 0.05 to 0.2 *M* were set up with 100 m*M* Tris pH 8.5 or 50 mM HEPES pH 7.5 and either 25% PEG 1000 or 10% PEG 3350.

Nano/microcrystals of CTB^AAAA^MPR were prepared by the ultrafiltration method. In this method, the supersaturated zone is reached by concentration of the protein by ultrafiltration while salt, precipitant and buffer concentrations remain constant. 300 µl purified protein (10 mg ml^−1^) was mixed with the same volume of precipitant solution consisting of 200 m*M* NH_4_HCO_2_, 30% PEG 3350, 10 m*M* CaCl_2_, 20 m*M* HEPES pH 7.5 in a 100 kDa cutoff concentrator (Amicon Microcon YM-100). The setup was then centrifuged to reduce the retentate volume by half to regain the original protein concentration of 10 mg ml^−1^. Following overnight incubation, more precipitant solution was added (30 µl) to further increase the yield of nano/microcrystals.

Crystallization conditions are summarized in Table 2 ▶.

### 2.6. Standard X-ray crystallography   

Characterization of the CTB^GPGP^MPR crystals was performed using synchrotron X-ray radiation on beamline 8.2.2 at the Advanced Light Source (ALS) at a wavelength of 1 Å. The crystals were flash-cooled in liquid nitrogen with a cryoprotectant solution (15% ethylene glycol, 50% PEG 400, 100 m*M* HEPES, 60 m*M* NaCl, 200 m*M* BaCl_2_, 150 m*M* imidazole, 0.017% βDDM) and diffraction data were collected at 100 K using an Oxford Cryostream. A total of 520 data frames were collected using 0.25° oscillations and an exposure time of 2.275 s per frame with an ADSC 315 detector.

### 2.7. Serial femtosecond nano/microcrystallography   

Nano/microcrystals were grown on-site and were analyzed by DLS prior to serial femtosecond X-ray nano/microcrystallography using the high-energy free-electron laser at the Coherent X-ray Imaging (CXI) endstation of the Linac Coherent Light Source (LCLS) at SLAC National Accelerator Laboratory (Experiment L432, February 2012). This method allows data to be collected from hundreds of thousands of sub-micrometre nano/microcrystals (by spraying them across a pulsed X-ray laser beam) using X-ray snapshots so brief that they outrun radiation damage (for a review of the method, see Spence *et al.*, 2012 ▶). Data were collected from a stream of fully hydrated nano/microcrystals. Experimental details of the beamline and data collection at CXI have been described by Boutet & Williams (2010 ▶) and Boutet *et al.* (2012 ▶). A suspension of nano/microcrystals of CTB^AAAA^MPR (9.1 mg ml^−1^, total volume of 330 µl) was supplied to the FEL X-ray beam using a gas-focused liquid microjet of 4 µm diameter at 20°C, a temperature-controlled antisettling device and a flow rate of 10 µl min^−1^ using a gas dynamic virtual nozzle (Weierstall *et al.*, 2012 ▶; DePonte *et al.*, 2008 ▶; Weierstall *et al.*, 2008 ▶; Lomb *et al.*, 2012 ▶). X-ray data were collected from the crystals at an energy of 6.3 keV with a 50 fs pulse duration and an X-ray pulse repetition rate of 120 Hz. Diffraction patterns from protein crystals were identified and selected using the hit-finding program *Cheetah* (Barty *et al.*, 2014 ▶), and indexing and merging was performed using *CrystFEL* (Kirian *et al.*, 2011 ▶; White *et al.*, 2012 ▶).

## 3. Results and discussion   

### 3.1. CTB^GPGP^MPR   

Previous work suggested that the immunogenicity of the MPR depends on its structural context, especially when fused to other proteins and peptides as is the case for CTB-MPR (Gach *et al.*, 2011 ▶; Montero *et al.*, 2012 ▶; Matoba *et al.*, 2008 ▶, 2011 ▶). Three different CTB-MPR fusion variants were designed that would differ in the linker peptide between the two fusion partners.

The original fusion protein that was described previously (Matoba *et al.*, 2004 ▶) contained a GPGP linker. It is denoted here as CTB^GPGP^MPR (Fig. 1 ▶ *b*). Two additional variants were created as part of the present study with the GPGP linker either deleted (CTBMPR; Fig. 1 ▶ *c*) or replaced by a tetra-Ala linker (CTB^AAAA^MPR; Fig. 1 ▶ *d*). To maximize expression levels in bacterial cells, all constructs reported here were devoid of a terminal histidine tag. Instead, we took advantage of a peculiarity of the CTB pentamer, preserved in the context of the fusion proteins, that allows it to specifically bind to metal-affinity resin (Dertzbaugh & Cox, 1998 ▶). Importantly, in the absence of a His tag only assembled pentamers can bind to the metal column (Dertzbaugh & Cox, 1998 ▶). The fusion proteins were expressed as described by Matoba *et al.* (2008 ▶) and were purified as described in §[Sec sec2]2 using the mild detergent βDDM for solubilization and in all further purification steps to facilitate crystallization efforts and biophysical analyses.

**Figure 2:**
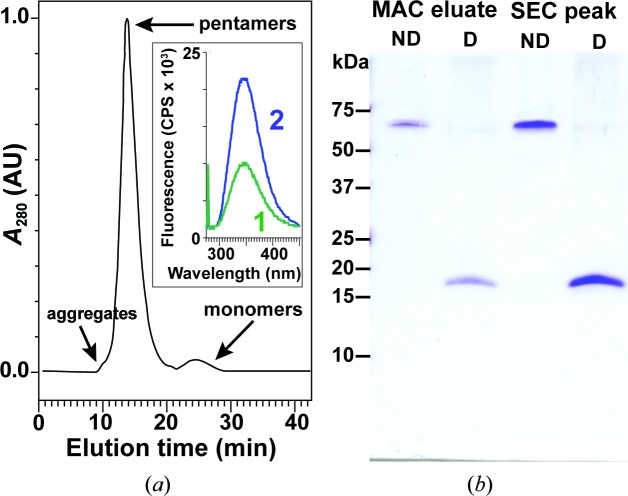
(*a*) Separation of aggregates and monomers from the pentameric CTB^GPGP^MPR protein by gel filtration on a Superdex 200 column. Assembly status was estimated from parallel resolution of molecular-mass standards (not shown). Inset, tryptophan fluorescence emission spectra of pentameric CTB^GPGP^MPR in pooled gel-filtration fractions corresponding to the major peak in (*a*). 1 (green), pentamers; 2 (blue), concentrated (Centricon 100) pentamers. Excitation was at 280 nm. (*b*) Proteins in the unconcentrated metal-affinity chromatography (MAC) eluate and in the size-exclusion chromatography (SEC) fraction corresponding to the main peak of the chromatogram in (*a*) were resolved by SDS–PAGE under nondenaturing (ND; no DTT and no boiling) and denaturing (D) conditions. Molecular-weight standards indicate that CTB^AAAA^MPR is organized into SDS-stable pentamers. The compact pentamers have a slightly higher electrophoretic mobility than expected based on their mass alone.

The purification scheme described above for CTB^GPGP^MPR fusion proteins resulted in >99% purity based on silver-stained polyacrylamide gels (Matoba *et al.*, 2008 ▶). As previously demonstrated by nondenaturing gel electrophoresis and by G_M1_ ganglioside ELISA (Matoba *et al.*, 2004 ▶, 2008 ▶), such protein preparations were highly homogeneous, consisting of primarily pentameric CTB^GPGP^MPR and only minor amounts of higher molecular-weight aggregates and monomeric protein. We were able to separate these various molecular forms by SEC–HPLC (Fig. 2 ▶ *a*). Oligomeric state assignment of the peaks was performed based on parallel SEC–HPLC runs with molecular-weight standards. This assignment was confirmed by resolving proteins in the pooled fractions corresponding to the peaks by SDS–PAGE under nonreducing conditions, which allows CTB to retain its pentameric organ­ization (Fig. 2 ▶ *b*; Yasuda *et al.*, 1998 ▶; Zrimi *et al.*, 2010 ▶). Taken together with the fact that that CTB^GPGP^MPR binds to the affinity resin, we conclude that the fusion protein is a stable pentamer.

Taking advantage of the presence of five tryptophan residues within the MPR domain (with one more within the CTB moiety), we subjected the proteins in the pooled fractions corresponding to CTB^GPGP^MPR pentamers to fluorescence spectroscopy (Fig. 2 ▶ *a*, inset). The emission spectrum revealed that the Trp residues in the pentamers were exposed to the aqueous milieu (peak emission at 347 nm; Ni *et al.*, 2011 ▶; Reshetnyak *et al.*, 2001 ▶). The stability of the pentamers was demonstrated by the conservation of the Trp emission profile upon purification and concentration of the protein.

We screened a large number of crystallization conditions which included systematic variation of the protein concentration, pH, precipitant and ionic strength. Furthermore, we tested the reversibility of the crystallization conditions. The initial screens provided important information on the solubility of CTB^GPGP^MPR. The addition of galactose is essential for crystallization of the protein, while only irreversible precipitation was observed in its absence. Reversible precipitation was observed at pH 7–8 and at medium salt concentrations (50–250 m*M*). Crystallization was favored by the addition of divalent cations (*e.g.* Ca^2+^) over monovalent cations, and shorter-chain polyethylene glycol polymers (PEGs) were the preferred precipitants.

We found multiple conditions where crystals formed (Supplementary Fig. S1). The crystals were grown in 0.1 *M* HEPES pH 7.5, 25–30% PEG 400, 0.2 *M* CaCl_2_, 0.3 *M* galactose, 80–100 m*M* NaCl at a protein concentration of 5 mg ml^−1^. The vapor-diffusion method (sitting drop) using ‘screw-cap’ plates (NeXtal) was used. Isolated crystals were cooled in liquid nitrogen in crystallization buffer containing 36% PEG 400 as a cryoprotectant. X-ray data were collected on beamline 8.3.1 at the Advanced Light Source (ALS). Most of the 50 µm crystals diffracted to about 20 Å resolution. The reflections were broad and anisotropic, indicative of the low order of the crystals in three dimensions. One unit-cell parameter was identified to be 45 Å.

**Figure 3:**
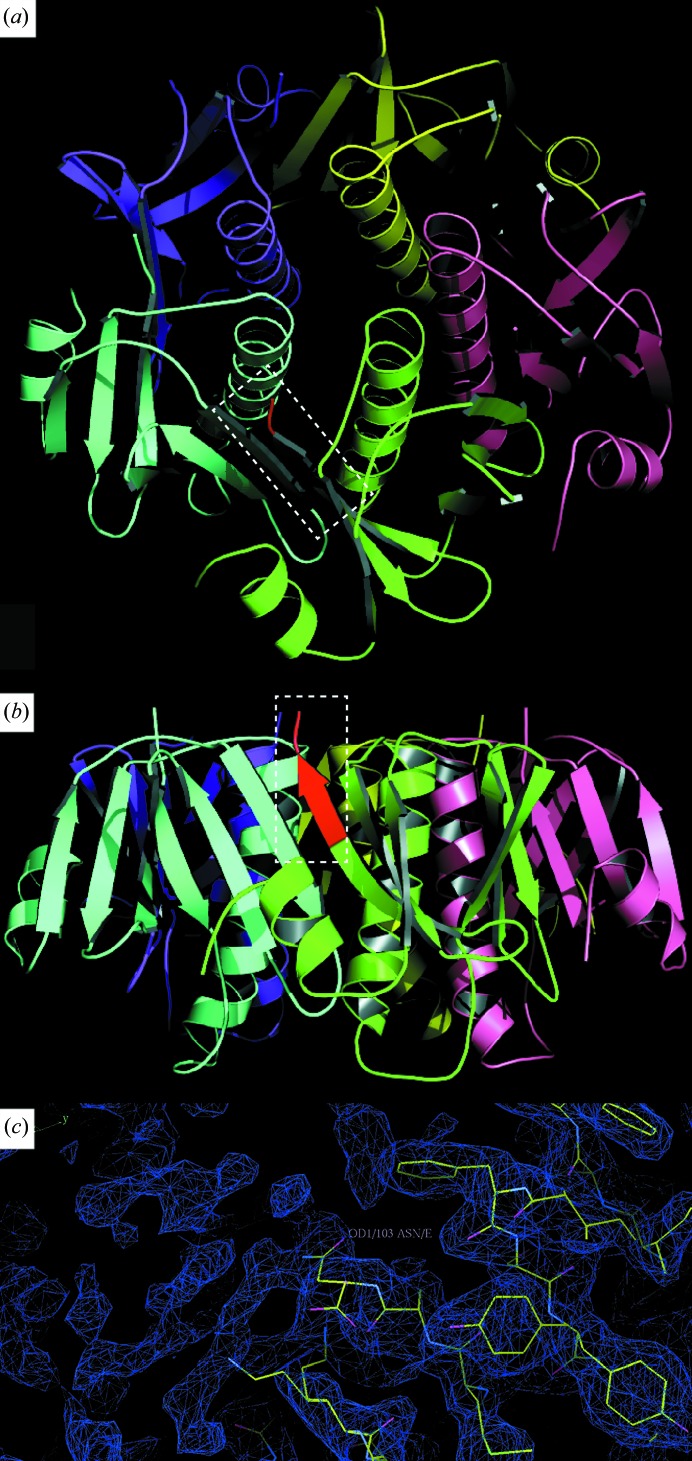
The CTB^GPGP^MPR structure reveals the expected pentameric ring arrangement typical of wild-type CTB but not the structure of the MPR. Cartoon representation of the crystal structure of CTB^GPGP^MPR in two orientations: (*a*) top view, (*b*) side view. Each subunit is indicated by a different color. The C-terminus of one of the subunits is indicated in red. This region is shown in close-up in (*c*). (*c*) 2*F*_o_ − *F*_c_ electron-density map at a contour level of 1.5σ of the C-terminus of CTB in CTB^GPGP^MPR, which was phased with the pentameric CTB model (PDB entry 1jr0; Pickens *et al.*, 2002 ▶) using molecular replacement (McCoy *et al.*, 2007 ▶). Electron density can be seen beyond the terminal asparagine of CTB where the GPGP linker and MPR connect.

Under slightly different crystallization conditions that included the presence of Zn^2+^ and lipids, crystals were observed that diffracted to a resolution limit of 2.3 Å. A full data set was collected from these crystals at the Advanced Photon Source (Table 3 ▶). Unfortunately, only the CTB region was ordered in the electron-density map, definitively demonstrating its pentameric nature (Figs. 3 ▶ *a* and 3 ▶ *b*). Weak electron density was observed that extended the C-terminus of CTB, but the structure of the MPR region could not be resolved in the crystals (Fig. 3 ▶ *c*). We hypothesized that this may be caused by the flexibility of the GPGP linker allowing the MPR region to assume multiple positions in the crystals.

### 3.2. CTBMPR   

To test our hypothesis regarding linker flexibility, we created a second fusion protein variant in which the movement of the MPR domain was expected to be restricted by direct fusion of the MPR to the C-terminus of the CTB protein (CTBMPR; Fig. 1 ▶ *c*).

**Figure 4:**
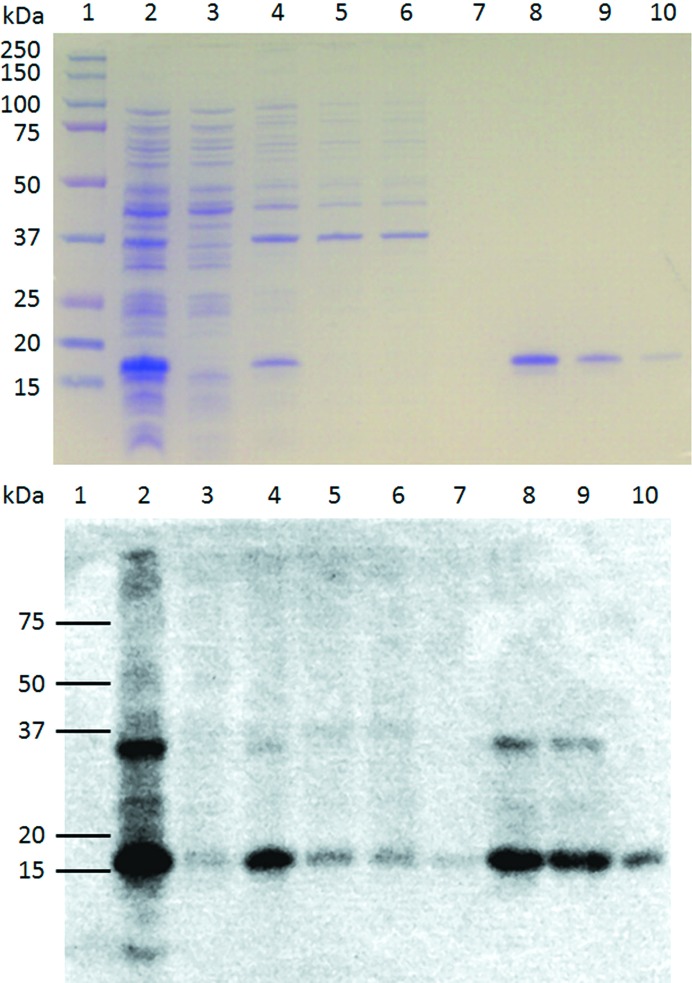
Affinity chromatography purification of CTBMPR. Protein samples from various steps in the purification process were resolved next to molecular-weight markers (lane 1) by SDS–PAGE and the gel was stained with Coomassie Brilliant Blue (upper panel). The whole cell lysate (lane 2) was spun down and the aqueous fraction (lane 3) was discarded. Membrane proteins were extracted from the pellet with βDDM (lane 4) purified over an affinity chromatography column. The flowthrough was collected (lane 5) and the column was extensively washed as described in the text (lane 6, first wash fraction; lane 7, last wash fraction). Elution required a larger volume of imidazole elution buffer to elute most of the protein bound to the column (lanes 8–10) than expected based on previous results with CTB^GPGP^MPR (Matoba *et al.*, 2008 ▶). Immuno­blotting was performed on the same samples using monoclonal 2F5 antibodies (lower panel).

The purification procedure for the linker-less fusion protein CTBMPR followed the same scheme as outlined above except that elution was conducted batchwise with extended incubation periods (from 10 min to 16 h) and higher concentrations of imidazole (300 m*M*) were required to elute most of the protein from the column (Fig. 4 ▶). The molecular mass of the fusion protein as estimated based on SDS–PAGE resolution (Fig. 4 ▶ *a*) and immunoblotting (Fig. 4 ▶ *b*) fitted the calculated value based on the sequence of the protein (17 kDa).

**Figure 5:**
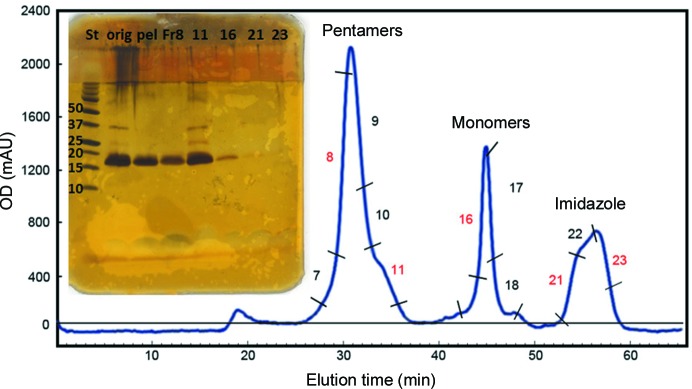
CTBMPR exists in several metastable oligomeric forms. Affinity-purified CTBMPR was resolved by SEC–HPLC, yielding three major peaks probably corresponding to pentamers (fraction 8) and monomers (fraction 16). Fractions 21 and 23 did not contain appreciable amounts of protein and are likely to contain high concentrations of imidazole. The shoulder at the right of the pentamer peak (fraction 11) may represent the less stable intermediates tetramers and dimers. These fractions (numbered in red), alongside the original sample and a precipitate that formed in the original sample, were analyzed by SDS–PAGE followed by silver staining (inset).

The homogeneity of the fusion protein in the pooled eluted fractions was tested by SEC–HPLC. This demonstrated that the preparation can be resolved into various peaks (Fig. 5 ▶). The results showed that unlike CTB^GPGP^MPR, the linker-less fusion protein exists in an equilibrium between several oligomeric molecular forms. Assignment of the oligomeric forms is based on the similarity in the elution volumes of the respective peaks to those of CTB^GPGP^MPR. Pentamers are not the predominant form of the linker-less CTBMPR protein, at least under our purification conditions. A substantial monomeric population is present alongside the pentamers in preparations obtained under similar purification conditions to those used in the purification of CTB^GPGP^MPR. In fact, since all of the protein loaded onto the SEC–HPLC column was specifically eluted from the metal-affinity column (and consequently must have been pentameric), it is likely that the CTBMPR pentamer undergoes (partial) disassembly during manipulation following the metal-affinity chromatography stage.

While gp41 is generally assumed to form trimers (Liu *et al.*, 2008 ▶; Atilgan *et al.*, 2010 ▶) in its pre-fusion form, the involvement of the MPR domain in trimerization is less clear and evidence for alternative associations exist (see, for example, Alfsen & Bomsel, 2002 ▶). This suggests that the equilibrium between the various oligomeric states is dynamic and may be explained by the competing tendencies of the CTB fusion partner to form pentamers, while the MPR fusion partner may push the equilibrium against pentamerization.

**Figure 6:**
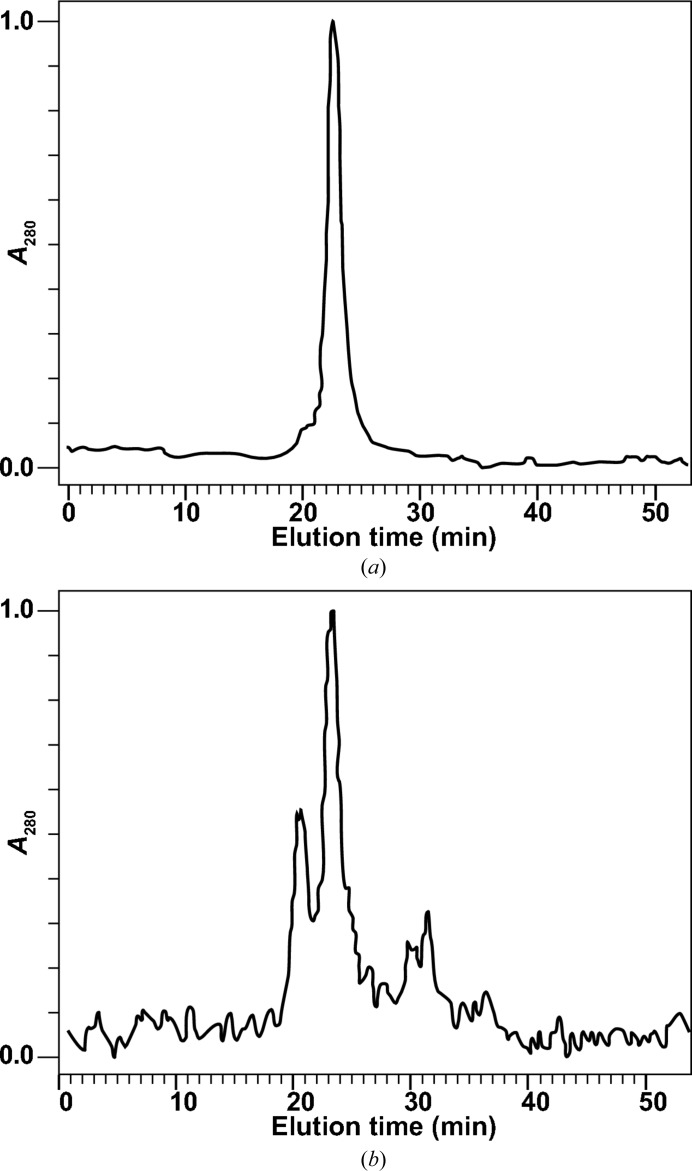
The CTBMPR oligomeric state is affected by the concentration of the protein. SEC–HPLC fractions corresponding to the pentamer (*a*) and monomer (*b*) peaks (Fig. 5 ▶) were subjected separately to a second SEC–HPLC purification. Absorbance is normalized to the highest peak.

To investigate this hypothesis, we separately pooled the fractions corresponding to the monomeric and the pentameric forms of CTBMPR, concentrated them and analyzed them separately by SEC–HPLC (Fig. 6 ▶). The pentamer appeared to be stable, leading to a single peak with the same elution time. However, upon concentration of the monomer-containing fractions, most of the fusion protein was shown to elute as a fraction corresponding to the pentamer fraction, suggesting a reorganization of the protein into pentamers. These results provided support for our hypothesis that a dynamic concentration-dependent equilibrium exists between the various oligomeric forms of CTBMPR, where lower concentrations favor monomers and higher concentrations favor pentamer formation.

We carried out crystallization experiments of CTBMPR using the vapor-diffusion method and broad crystal screening, as described earlier, to identify conditions where crystals were able to form. Disappointingly, only a few conditions led to ordered precipitate or pseudo-crystals, and finer screens around the conditions did not produce three-dimensionally ordered crystals. A possible explanation is that the instability of the oligomeric states hinders the formation of crystals.

### 3.3. CTB^AAAA^MPR   

**Figure 7:**
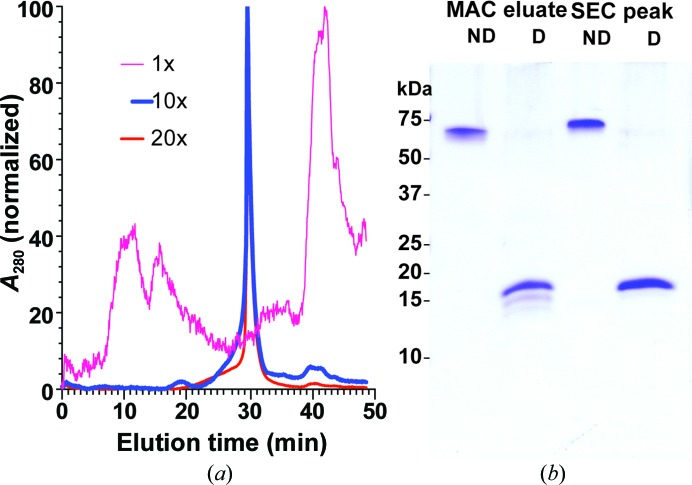
CTB^AAAA^MPR resolved as an oligomer by SEC–HPLC. Pink line, the Talon column eluate (not concentrated). Blue line, 10× concentrated eluate sample. Red line, 20× concentrated eluate sample. Spectrograms were normalized to the highest peak. Inset: proteins in fractions corresponding to the main peak of the 20× concentrated eluate chromatogram were resolved by SDS–PAGE under nondenaturing (ND; no DTT and no boiling) and denaturing (D) conditions. Molecular-weight standards indicate that CTB^AAAA^MPR is organized into SDS-stable pentamers.

Based on the results with CTBMPR, we designed a third variant of the CTB-MPR fusion protein, CTB^AAAA^MPR (Fig. 1 ▶ *d*), that links the two fusion partners with a short polyalanine peptide that is expected to assume an α-helical conformation (O’Neil & DeGrado, 1990 ▶). Our aim was to allow the fusion protein to assemble into stable pentamers by facilitating the ability of the MPR moieties to interact with each other while avoiding presumed disorder induced by the flexible GPGP linker. The SEC–HPLC purification profile resembled that for the CTB^GPGP^MPR variant (Fig. 7 ▶ *a*). The formation of the pentamer, as verified by nondenaturing SDS–PAGE, was still concentration-dependent; however, the pentamer was much more stable for CTB^AAAA^MPR than for the linker-less construct CTBMPR (Fig. 7 ▶ *b*).

**Figure 8:**
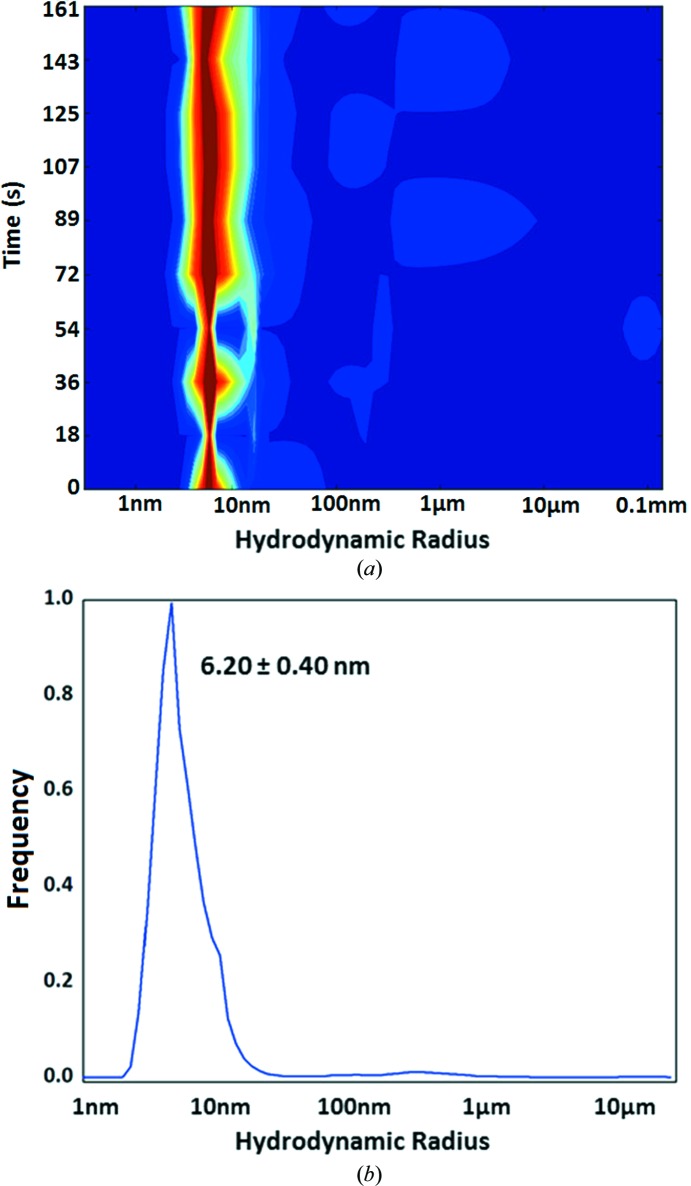
CTB^AAAA^MPR is monodisperse as a high-order oligomer. (*a*) DLS measurements were performed so that the size distribution in the sample was analyzed for 20 s and the measurement was repeated consecutively ten times. The moment-to-moment fraction of particles estimated to have a particular hydrodynamic radius is color-coded and shown as a heat plot (red, >90%; blue, none). The narrow vertical and red profile shown indicates high stability over the duration of the measurement and low polydispersity. Time: the total duration of the scanning session (200 s). (*b*) A distribution curve of particle-size frequencies gives a more quantitative evaluation of the polydispersity, with the mean ± SD indicated next to the peak. The standard deviation of the size distribution is only 6% of the mean, indicating low polydispersity.

We obtained the size distribution of the purified CTB^AAAA^MPR by dynamic light scattering (DLS) to determine whether the protein preparation was monodisperse (Fig. 8 ▶). At 8 mg ml^−1^, the hydrodynamic radius (Stokes radius, *r*) of the detergent-solubilized protein (*i.e.* of the protein–detergent micelles) was determined to be 6.2 ± 0.4 nm. The polydispersity was estimated to be 6%, which is well below the 10–15% level considered as monodisperse (Proteau *et al.*, 2010 ▶). Note that the DLS measurement in Fig. 8 ▶ shows the direct scattering intensity, which is not corrected for the molecular mass of the particles to detect even traces of aggregates. As the increase in scattered intensity is proportional to *r* ^6^, we calculated that the sample was highly monodisperse and contained less than 0.00001% aggregates. Since the exact geometry of CTB^AAAA^MPR is not known, a generic set of parameters was used assuming that the folded state is spherical with an estimated molecular mass of ∼210 kDa, which includes the detergent bound to the protein. The DLS data indicated that CTB^AAAA^MPR may form a dimer of pentamers, corresponding to a molecular weight of 170 kDa for the protein, while a trimer of pentamers would be 250 kDa larger than the value calculated based on the DLS results. However, it is difficult to determine how much of the estimated molecular mass was associated with the detergent micelles around the hydrophobic regions of the protein.

**Figure 9:**
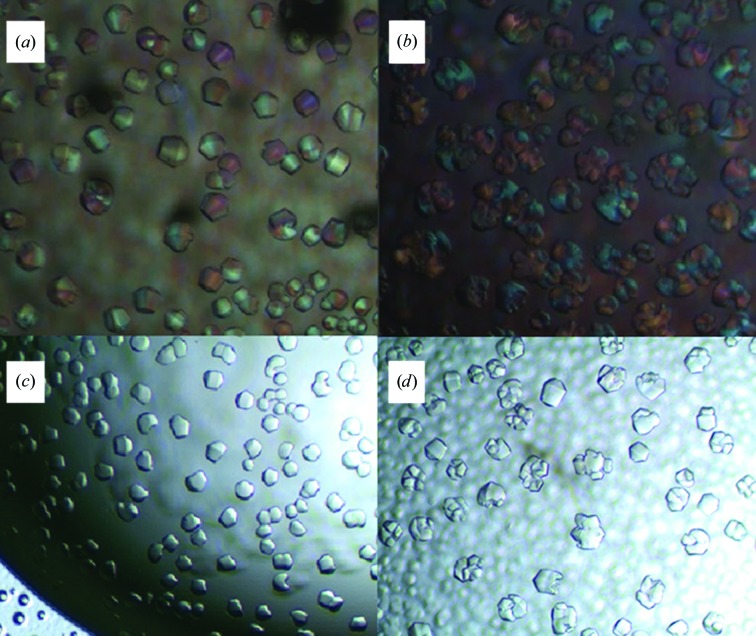
CTB^AAAA^MPR crystals form under different conditions of a fine screen. (*a*) 0.2 *M* ammonium formate, 8% PEG 3350. (*b*) 0.2 *M* ammonium formate, 5% PEG 3350. (*c*) 0.2 *M* ammonium formate, 12% PEG 3350. (*d*) 0.1 *M* ammonium formate, 10% PEG 3350

A large set of crystallization experiments was carried out with purified CTB^AAAA^MPR similarly to that described above for the linker-less variant CTBMPR. Crystals were observed more frequently for CTB^AAAA^MPR than for CTBMPR, but despite the fact that CTB^AAAA^MPR appeared to be more stable and more homogeneous than CTBMPR, the crystal quality was still poor. Under most conditions, pseudo-crystals were observed and were similar in shape to the CTB^GPGP^MPR crystals (Fig. 9 ▶). The crystals shown in Fig. 9 ▶(*a*) feature a hexagonal shape when viewed from the ‘top’, but are completely round when viewed from the side. X-ray diffraction patterns from these crystals show features of a hexagonal powder diffraction pattern, which may indicate that the crystals consist of stacks of two-dimensional crystals which are disordered in the third dimension. However, we noticed that crystal disorder seemed to be correlated with the size of the crystals, with larger crystals displaying more disorder.

**Figure 10:**
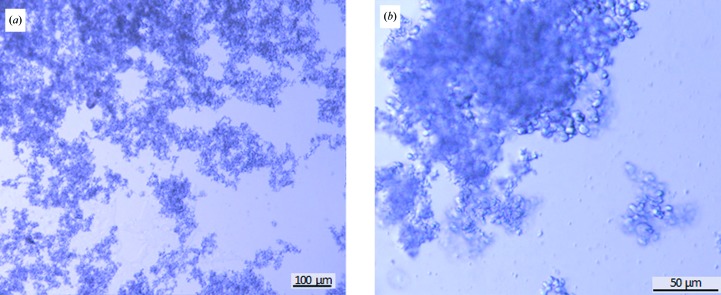
Nano/microcrystals of CTB^AAAA^MPR grown in 0.2 *M* ammonium formate, 30% PEG 3350 before (*a*) and after (*b*) filtering through a 20 µm filter. The cystals in (*b*) are shown at a higher magnification.

**Figure 11:**
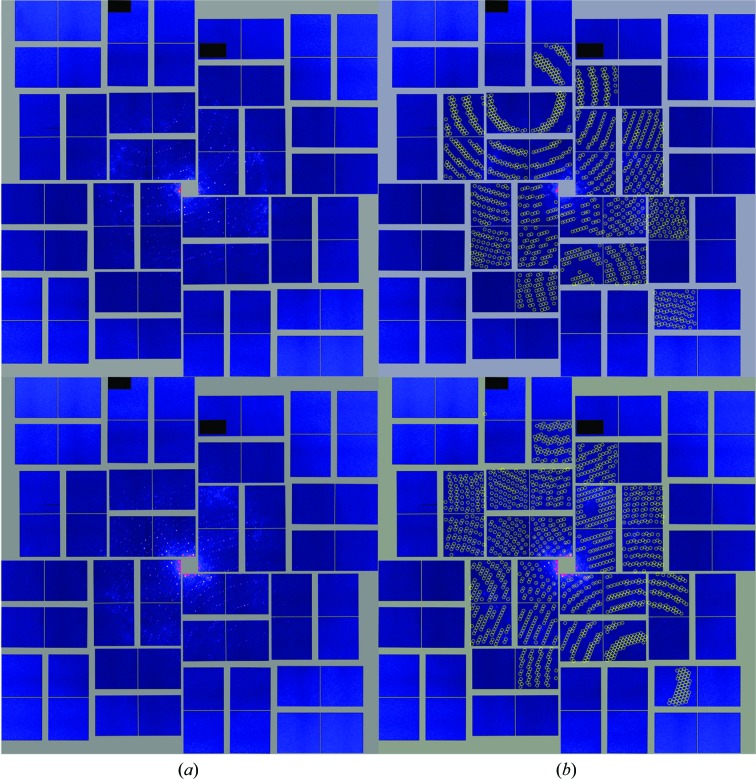
(*a*) Two CTB^AAAA^MPR diffraction patterns collected from nano/microcrystals on the CXI beamline at LCLS in February 2012. (*b*) Indexing of the diffraction patterns in (*a*). The yellow circles indicate the predicted positions of the reflections.

Taking this into account, crystals were rapidly grown by a fast increase of the supersaturation state using ultrafiltration to concentrate the protein at a constant precipitant concentration (Fig. 10 ▶). Most of the crystals were smaller than the shortest wavelength of visible light; they had the appearance of amorphous precipitates, with very small microcrystals also visible in the sample (Fig. 10 ▶), and this mixture of small (1–2 µm) and very small (<1 µm) crystals will be referred to here as ‘nano/microcrystals’. CTB^AAAA^MPR nano/microcrystals were grown on site at LCLS, characterized by DLS and SONICC and their diffraction quality was tested by the new method of serial femtosecond crystallography (SFX) on the CXI beamline at the LCLS. This beamtime was dedicated to the exploration of the use of SFX for structure elucidation of membrane proteins following the seminal work by Chapman *et al.* (2011 ▶) and Boutet *et al.* (2012 ▶). These articles provide detailed description of sample delivery and data collection that will only briefly be recounted here (see the review by Spence *et al.*, 2012 ▶). Millions of X-ray data diffraction snapshots were collected from a stream of protein nanocrystals or microcrystals in their mother liquor at room temperature as they flow across the beam. Diffraction snapshots of individual crystals of CTB^AAAA^MPR were collected using X-rays pulses of extremely high intensity (10^9^ higher peak brilliance than the brightest third-generation synchrotrons). The 10–50 fs pulses are so brief that the diffraction of each nano/microcrystal is recorded before it is disintegrated. This diffract-before-destroy principle (Barty *et al.*, 2012 ▶) overcomes the X-ray damage problem in conventional crystallography and allows data collection from crystals that contain only a few hundred molecules (Chapman *et al.*, 2011 ▶). The results from the LCLS beamtime were very promising, as we were able to grow crystals on site and detected the first single-crystal diffraction patterns from CTB^AAAA^MPR nano/microcrystals. While the larger crystals of CTB^AAAA^MPR were disordered in the third dimension, the nano/microcrystals are ordered in all three dimensions and show a low degree of disorder. We did not observe any anisotropy of the diffraction patterns even in the third dimension. This is particularly striking since the nano/microcrystals of the protein were grown using the same set of precipitants at initial higher concentration, therefore reaching the supersaturation and nucleation phase much faster than in the vapor-diffusion experiment leading to the larger dis­ordered crystals. A single sort short run of the CTB^AAAA^MPR nano/microcrystals allowed us to collect 1006 patterns, most of which showed diffraction to 4–6 Å resolution and were successfully indexed (see two typical diffraction patterns and their indexed images in Fig. 11 ▶; Table 4 ▶). From the indexed patterns, we were able to determine the space group and the unit-cell parameters. The crystals appear to be rhombohedral (consistent with point group *R*32 with unit-cell parameters *a* = *b* = *c* = 332 Å, α = β = γ = 60°). There are only a few published examples of structures with space group *R*3 and a similar unit-cell parameter to that we observed here for the CTB^AAAA^MPR fusion protein. Interestingly, the three examples we could find in the PDB happen to be of viral origin. These PDB entries include the structure of *Physalis mottle virus* (PDB entry 1qjz; Krishna *et al.*, 1999 ▶), with unit-cell parameters *a* = *b* = *c* = 294 Å, α = β = γ = 59.91°, and the structures of the *Sesbania mosaic virus* coat protein (PDB entry 1smv; Bhuvaneshwari *et al.*, 1995 ▶) and its mutant (PDB entry 1x33; Sangita *et al.*, 2005 ▶), with unit-cell parameters *a* = *b* = *c* = 291 Å, α = β = γ = 62°.

Since each diffraction pattern is a ‘still image’ and most reflections are partial, accurate determination of structure requires high redundancy of the data set, *i.e.* many recordings in the vicinity of each reflection, in order to provide angular integration across the Bragg condition. For example, the first near-atomic resolution structure of a protein to be determined using femtosecond crystallography contained more than 12 000 indexed diffraction patterns (Boutet *et al.*, 2012 ▶). While the minimum number of single crystal hits that are required for structure analysis is currently unknown, the thousand reflections that we were able to collect with our very small sample size did not constitute a full native data set that could support structure determination; more data will have to be collected to this end.

It was surprising to see that the nano/microcrystals of CTB^AAAA^MPR (most of which are <1 µm) are ordered in three dimensions while the larger (100–300 µm) crystals grown with the same set of precipitants are completely disordered in the third dimension. We are currently screening conditions and applying seeding techniques to grow crystals of defined micrometre sizes from the nano/microcrystals for conventional X-ray data collection at synchrotron microfocus beamlines. The plan is to test the diffraction quality of crystals with target sizes ranging from 5 to 100 µm to determine up to which size the crystals are still ordered in three dimensions, with the goal of identifying a ‘single-crystal threshold’ that may enable data collection at microfocus beamlines. We can then further optimize the crystal quality of the microcrystals by fine screening of the conditions, including the screening of additives.

NMR and crystal structures have been determined of small peptide derivatives of the MPR region that contain binding sites for neutralizing antibodies (Biron *et al.*, 2005 ▶; Song *et al.*, 2009 ▶; Pejchal *et al.*, 2009 ▶) and the consensus is that this peptide can assume an α-helical conformation. Further structural information on the MPR region was obtained by studies involving an *in vitro*-assembled six-helix bundle consisting of separately produced peptide derivatives of gp41 (Shi *et al.*, 2010 ▶) and a chimeric protein consisting of a series of gp41 peptides separated by linkers (Buzon *et al.*, 2010 ▶). The conformations observed in these studies are very likely to represent the post-fusion form of MPR. However, the structure shows that the 2F5 binding site is deeply buried inside the three-helix bundle (Shi *et al.*, 2010 ▶; Buzon *et al.*, 2010 ▶) and therefore these constructs may not induce 2F5-like neutralizing antibodies. During the fusion process, large conformational changes must occur in gp41 that break the interaction between the trimers and expose the 2F5 antibody-binding site, thereby allowing 2F5 to block fusion and transcytosis; thus, a structure of the fusion-active form of MPR is highly desired. Our ultimate goal is to design an optimal CTB-MPR construct that can serve as a vaccine against HIV. Our design of the MPR fusion with CTB is based on the idea of a symmetry mismatch, where the pentameric oligomeric state of CTB hinders the formation of trimers of MPR and thereby stabilizes the MPR region of gp41 in its pre-fusion active form. While we present major strides in this work, further improvement of both the traditional X-ray crystallography approach (including co-crystallization with neutralizing antibodies) and less-explored innovations such as serial femto­second crystallography are needed to allow us to meet this goal.

This work presents a proof of principle that three-dimensionally ordered nano/microcrystals can be grown from a protein that had so far resisted growth of any macroscopic crystals that were ordered in three dimensions. Most remarkable is the fact that the SFX diffraction patterns clearly indicate that the nano/macrocrystals were single crystals, while macroscopic crystals grown with the same chemical compounds as precipitants showed the features of two-dimensional crystals stacked nearly randomly in the third dimension. Further enhancement of the quality of the nano/microcrystals by application of improved methods of nanocrystal growth (Kupitz *et al.*, 2014 ▶) and the collection of a full data set from these crystals by serial femtosecond nano­crystallography would allow us to determine the structure of CTB^AAAA^MPR.

## Supplementary Material

Supplementary Figure S1.. DOI: 10.1107/S2052252514014900/mf5003sup1.pdf

## Figures and Tables

**Table 1:** Oligonucleotides used as primers in this study

No.	Name	5′-Sequence-3′
1	oTM066	AGCCATGGGCACCCCACAAAACATCACTG
2	oTM123	ATTGCTCAGCGGTTCAGATCTTGATATACCAAAGC
3	oTM468	GGCAAATTCCCAAACCCAACAAGAGAAGAATG
4	oTM469	CTTGTTGGGTTTGGGAATTTGCCATGCTAATGGCAGC
5	oTM521	GCGGCCGCGGCCTCCCAAACCCAACAAGAG
6	oTM522	GGCCGCGGCCGCATTTGCCATGCTAATGGC

**Table 2:** Crystallization conditions

Construct	Conditions
CTB^GPGP^MPR	34% PEG 400, 0.2 *M* BaCl_2_, 20% ethylene glycol, 0.5 *M* ammonium acetate, 0.74% CYMAL-4
	25–30% PEG 400, 0.2 *M* CaCl_2_, 0.1 *M* HEPES pH 7.5, 0.3 *M* galactose, 80–100 m*M* NaCl
CTBMPR	25–30% PEG 300, 0.2 *M* CaCl_2_, 0.05 *M* HEPES pH 7.5, 0.02% βDDM
	25–30% PEG 300, 0.2 *M* NaCl, 0.05 *M* HEPES pH 7.5, 0.02% βDDM
	25–30% PEG 300, 0.2 *M* NH_4_Cl, 0.05 *M* HEPES pH 7.5, 0.02% βDDM
CTB^AAAA^MPR	8–12% PEG 3350, 0.1–0.2 *M* NH_4_HCO_2_, 0.01 *M* CaCl_2_, 0.05 *M* HEPES pH 7.5, 0.02% βDDM
CTB^AAAA^MPR nano/microcrystals	30% PEG 3350, 0.2 *M* NH_4_HCO_2_, 0.01 *M* CaCl_2_, 0.05 *M* HEPES pH 7.5, 0.02% βDDM

**Table 3:** Crystallographic data for CTB^GPGP^MPR Values in parentheses are for the highest resolution bin.

Wavelength (Å)	1.0
Resolution range (Å)	59.48–2.10 (2.21–2.10)
Space group	*R*3:*H*
Unit-cell parameters (Å, °)	*a* = *b* = 174.39, *c* = 64.71, α = β = 90, γ = 120
Total reflections	162139
Unique reflections	42785
Multiplicity	3.8 (3.8)
Completeness (%)	99.95 (100.00)
Mean *I*/σ(*I*)	6.68 (1.93)
Wilson *B* factor (Å^2^)	30.72
*R*_merge_	0.136 (1.302)
*R* factor	0.214 (0.315)
*R*_free_	0.249 (0.388)
No. of atoms	4365
No. of macromolecules	4100
No. of waters	265
No. of protein residues	515
R.m.s.d., bonds (Å)	0.008
R.m.s.d., angles (°)	1.08
Ramachandran favored (%)	98
Ramachandran allowed (%)	1.8
Ramachandran outliers (%)	0.2
Clashscore	8.52
*B* factors (Å^2^)	
Average	40
Macromolecules	39.9
Solvent	42.6

**Table 4:** Crystallographic data for CTB^AAAA^MPR

Run time	10 min 40 s
Total No. of raw frames	72767
No. of crystal hits	1006
Hit rate (%)	1.38
No. of indexed patterns	55
Indexing yield (%)	5.46
Unit-cell parameters (Å, °)	*a* = *b* = *c* = 332, α = β = γ = 60
Space group	*R*32
