# Supplementary material for: Expression, purification and crystallization of CTB-MPR, a candidate mucosal vaccine component against HIV-1
Source: IUCrJ. 2014 Aug 20;1(Pt 5):305–17. doi: 10.1107/S2052252514014900 (PMC4174873; doi:10.1107/S2052252514014900)

IUCrJ

Volume 1 (2014)

Supporting information for article:

Expression, purification and crystallization of CTB-MPR, a candidate mucosal vaccine component against HIV-1

Ho-Hsien Lee, Irene Cherni, HongQi Yu, Raimund Fromme, Jeffrey D. Doran, Ingo Grotjohann, Michele Mittman, Shibom Basu, Arpan Deb, Katerina Dörner, Andrew Aquila, Anton Barty, Sébastien Boutet, Henry N. Chapman, R. Bruce Doak, Mark S. Hunter, Daniel James, Richard A. Kirian, Christopher Kupitz, Robert M. Lawrence, Haiguang Liu, Karol Nass, Ilme Schlichting, Kevin E. Schmidt, M. Marvin Seibert, Robert L. Shoeman, John C. H. Spence, Francesco Stellato, Uwe Weierstall, Garth J. Williams, Chunhong Yoon, Dingjie Wang, Nadia A. Zatsepin, Brenda G. Hogue, Nobuyuki Matoba, Petra Fromme and Tsafrir S. Mor

Figure S1 Gallery of crystals of CTB^{GPGP}MPR. The crystals were grown in 25-30% PEG400, 0.2 M $CaCl_2$, 0.1 M HEPES pH7.5, 0.3 M galactose, 80-100 mM NaCl at a protein concentration of 5mg/ml. The shape of the crystals indicates that they may be disordered in the third dimension as they usually have sharp corners on one view plain, whereas they appear round when viewed from the perpendicular side

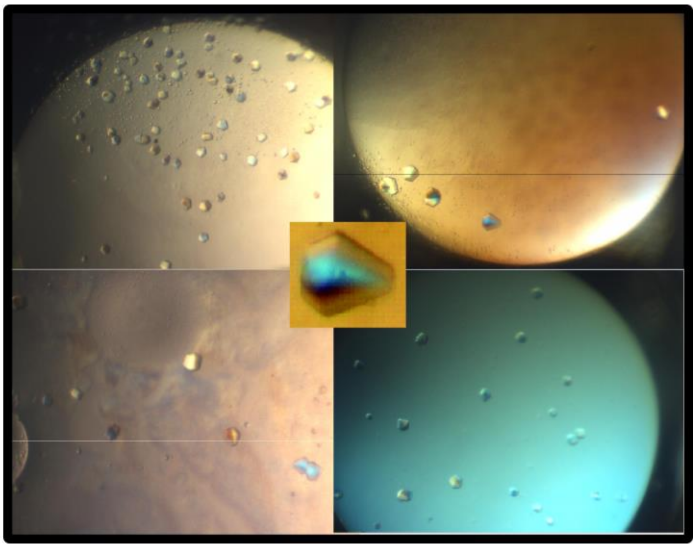

Supplement: Supplementary file 1 [file m-01-00305-sup1.pdf]
